# Stress affects theta activity in limbic networks and impairs novelty-induced exploration and familiarization

**DOI:** 10.3389/fnbeh.2013.00127

**Published:** 2013-10-14

**Authors:** Luis R. Jacinto, Joana S. Reis, Nuno S. Dias, João J. Cerqueira, José H. Correia, Nuno Sousa

**Affiliations:** ^1^Life and Health Sciences Research Institute, University of Minho, Campus de GualtarBraga, Portugal; ^2^ICVS/3B's – PT Government Associate LaboratoryBraga/Guimarães, Portugal; ^3^Department of Industrial Electronics, University of Minho, Campus de AzurémBraga, Portugal; ^4^DIGARC, Polytechnic Institute of Cavado and AveBarcelos, Portugal

**Keywords:** stress, anxiety, local field potentials, ventral hippocampus, amygdala, pre-frontal cortex

## Abstract

Exposure to a novel environment triggers the response of several brain areas that regulate emotional behaviors. Here, we studied theta oscillations within the hippocampus (HPC)-amygdala (AMY)-medial prefrontal cortex (mPFC) network in exploration of a novel environment and subsequent familiarization through repeated exposures to that same environment; in addition, we assessed how concomitant stress exposure could disrupt this activity and impair both behavioral processes. Local field potentials (LFP) were simultaneously recorded from dorsal and ventral hippocampus (dHPC and vHPC, respectively), basolateral amygdala (BLA) and mPFC in freely behaving rats while they were exposed to a novel environment, then repeatedly re-exposed over the course of 3 weeks to that same environment and, finally, on re-exposure to a novel unfamiliar environment. A longitudinal analysis of theta activity within this circuit revealed a reduction of vHPC and BLA theta power and vHPC-BLA theta coherence through familiarization which was correlated with a return to normal exploratory behavior in control rats. In contrast, a persistent over-activation of the same brain regions was observed in stressed rats that displayed impairments in novel exploration and familiarization processes. Importantly, we show that stress also affected intra-hippocampal synchrony and heightened the coherence between vHPC and BLA. In summary, we demonstrate that modulatory theta activity in the aforementioned circuit, namely in the vHPC and BLA, is correlated with the expression of anxiety in novelty-induced exploration and familiarization in both normal and pathological conditions.

## Introduction

Exposure to novelty triggers complex neural mechanisms that involve attention, arousal, and anxiety-driven defensive behaviors, including risk assessment (Grey and McNaughton, 2003; Silman and Soreq, 2004). These emotional behaviors are regulated by the interactions between several highly interconnected brain regions; amongst these are the hippocampus (HPC), the medial prefrontal cortex (mPFC), and the amygdala (AMY) which appear to form a unified circuit with a preponderant role in emotional behavior. The HPC, more specifically through its ventral component (vHPC), projects directly to both mPFC and AMY (Pitkänen et al., 2000). The AMY also receives massive projections from the mPFC (Orsini et al., 2011), and projects back to the mPFC where excitatory and inhibitory inputs from the HPC and AMY interact (Ishikawa and Nakamura, 2003). Theta oscillations (4–12 Hz) can provide temporal circuit coordination (Buzsáki, 2002), namely within the HPC-AMY-mPFC network, and, as a consequence, have been correlated with the expression of emotional behavior (Seidenbecher et al., 2003; Adhikari et al., 2010; Popa et al., 2010; Lesting et al., 2011). Previous studies have shown theta coupling variations between these regions associated with precise fear-eliciting stimuli (Seidenbecher et al., 2003; Lesting et al., 2011) but global theta activity variations underlying fear consolidation (Popa et al., 2010) and anxiety (Adhikari et al., 2010) have also been described. Although some of this studies (Gordon et al., 2005; Adhikari et al., 2010) have focused attention on theta oscillations and anxiety behavior in anxiety-provoking arenas; the effects of familiarization through repeated exposures to the same environment on these oscillations is not yet known, nor the role of the AMY in the observed responses.

Stress is a powerful modulator of emotional behavior (Pêgo et al., 2008; Bessa et al., 2009). Stress-induced effects on emotional behavior can have an adaptive value (McEwen, 2007) allowing precisely the coping with the stress response-eliciting events; however, chronic or prolonged exposure to stress may lead to a maladaptive response accompanied by dysfunctional behavioral responses (Sousa and Almeida, 2012). Interestingly, chronic stress is known to trigger structural and functional changes precisely in the HPC-AMY-mPFC network, albeit in opposite directions: while there is atrophy and decreased activation of the dorsal hippocampus (dHPC) and mPFC, the vHPC, and AMY become hypertrophic and hyperactive after prolonged stress exposure (Vyas et al., 2002; Cerqueira et al., 2007). The fact that this circuit is preferentially targeted by chronic stress helps understanding why the emotional domain has a preponderant outcome in stress-related disorders. More specifically, in terms of anxiety, chronic stress has been shown to induce a state of hyper-anxiety in rodents characterized by a higher sensitivity to anxiety-provoking stimuli (Pêgo et al., 2008). To the best of our knowledge no previous studies have analyzed theta activity in a freely moving rodent's model of chronic stress.

Exposure to a novel environment is a challenging situation that elicits anxiety-driven defensive responses (Blanchard et al., 2011). However, if exposures to the same environment become repetitive, and there are no salient threats that can be perceived as dangerous in it, familiarization will take place (Blanchard et al., 2011); importantly, this behavioral habituation is likely regulated by the modulation of the vHPC-AMY-mPFC circuit which is implicated in order to generate appropriate anxiety-driven defensive behaviors including risk assessment. Since theta activity within the HPC-AMY-mPFC circuit has been correlated with emotional orienting responses it was of interest to analyze theta activity within the circuit upon exposure to a novel environment and how the activity varied during the familiarization process. Therefore, the activity in the theta range of the HPC-AMY-mPFC circuit in local field potentials (LFP) recorded in the dHPC and vHPC, the basolateral amygdala (BLA) and the mPFC in freely behaving rats first exposed to a novel arena was longitudinally analyzed; this activity was then followed during familiarization with the same arena by repeated exposures over the course of 3 weeks and finally after re-exposure to another unfamiliar arena. Taking into account the effects of chronic stress on the same circuit, it was also of interest to observe how concomitant stress exposure could disrupt the activity of this network during familiarization to the arena and correlate with emotional disturbances when facing a novel challenge.

## Results

In order to study the effects of exposure to a novel environment and subsequent familiarization with that same environment in theta activity from the HPC-AMY-mPFC network, control rats (*n* = 7) were initially exposed to an unfamiliar open field (OF) arena and allowed to freely explore it. During the course of 3 weeks (21 days) following the initial exposure rats were re-exposed to the same environment twice a week. An additional group of rats (*n* = 7) was subjected to a chronic unpredictable stress (CUS) protocol during the same time period (20 days) on which the exposures to the OF arena took place.

### Efficacy of the stress protocol and behavioral impact

**Figure 1:**
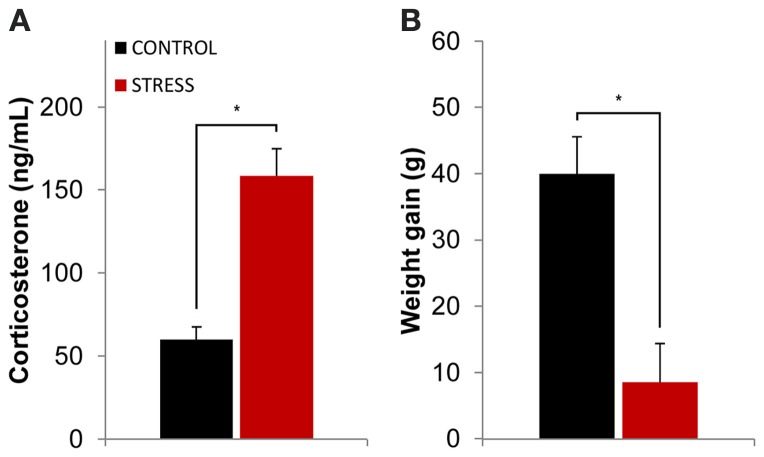
**Efficacy of the stress protocol. (A)** Plasmatic corticosterone levels measured on the day following the final exposure to the OF arena (which also corresponded to the day after the stress protocol ended). Stressed animals showed higher levels of plasmatic corticosterone than controls at the end of the stress protocol. **(B)** Body weight gain measured as the difference between the animal's weight at the beginning and ending of the stress protocol for control and stressed animals. Stressed animals showed higher levels of plasmatic corticosterone than controls at the end of the stress protocol as well as reduced body weight gain. ^*^p < 0.05 for unpaired Wilcoxon rank sum test comparison of average plasmatic corticosterone and body weight gain between control and stress groups. Error bars: ± s.e.m.

The efficacy of the stress protocol was evaluated through serum corticosterone measurements after the stress protocol ended (see methods section for details) and through the comparison of body weight between the beginning and ending of the stress protocol. Stressed animals showed higher levels of serum corticosterone than controls at the end of the stress protocol (*p* = 0.0001, Figure 1A). The stress protocol also slowed down body weight gain in stressed animals when compared to controls during the same time period (*p* = 0.005, Figure 1B).

**Figure 2:**
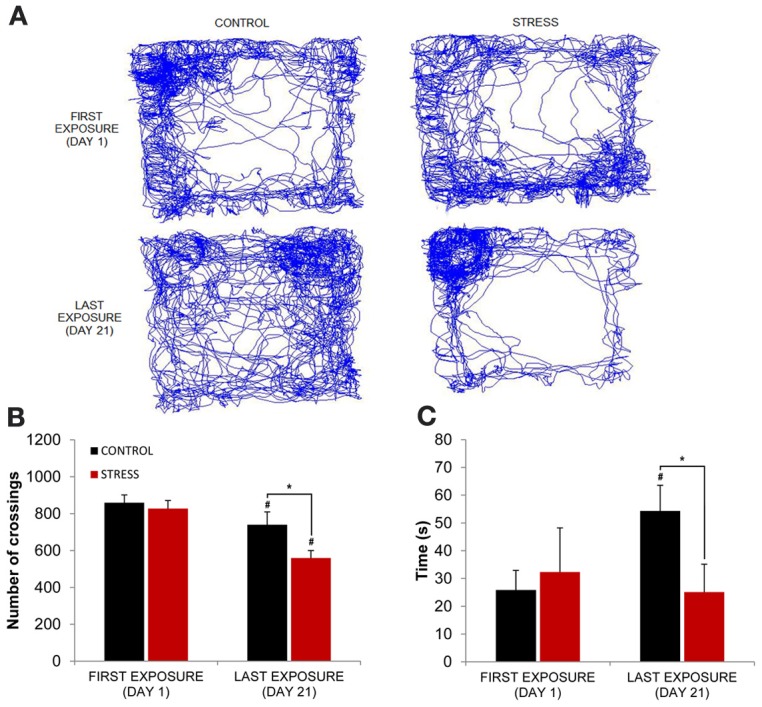
**Evolution of behavioral parameters through familiarization from first and last exposures to the OF arena. (A)** Representative trajectories from one rat from each group (control left; stress right) between the first (top) and last (bottom) exposures. **(B)** Comparison of exploratory activity, as measured by the total number of crossings between the squares on which the arena was divided for behavioral analysis, between first (left) and last (right) exposure to the OF arena on control (black bar) and stress (red bar) groups. **(C)** Comparison of time spent in the center of the OF arena between first (left) and last (right) exposure on control (black bar) and stress (red bar) groups. Rats from both groups significantly decreased exploratory activity between the first and last exposures to the arena, although the observed decrease was five times greater in stress group animals. Control rats significantly increased the time spent in the center of the arena from the first to the last exposure while stressed rats did not. #*p* < 0.05 for paired Wilcoxon sign rank test comparison of behavioral parameters within the same group between exposures; ^*^p < 0.05 for unpaired Wilcoxon rank sum test comparison of behavioral parameters between groups on each exposure. Error bars: ± s.e.m.

In terms of behavior, in the first day of exposure to the novel OF arena animals from both groups presented similar exploratory patterns (Figure 2A) with similar average distance travelled (as measured by the number of total crossings) (Figure 2B) and similar average time spent in the center (Figure 2C). In contrast, in the last day of exposure to the then familiar arena, control rats, on one hand, presented a significant increase of time spent in the center (*p* = 0.03, Figure 2C) while significantly reducing the average distance travelled (*p* = 0.03, Figure 2B) when compared with the first exposure. Stressed animals, on the other hand, presented a significant reduction of the average distance travelled (*p* = 0.01; last day stress vs. last day control *p* = 0.03, Figure 2B) and similar time spent in the center (last day stress vs. last day control *p* = 0.02, Figure 2C) when compared with the first exposure. It is important to note, however, that the reduction in average distance travelled between the first and last day of exposure to the arena in control animals was of small magnitude and the one observed in stressed rats was five times higher than the one observed for controls (stress 32% vs. control 6%).

### Familiarization with an environment is accompanied by theta power reduction in the vHPC and BLA

**Figure 3:**
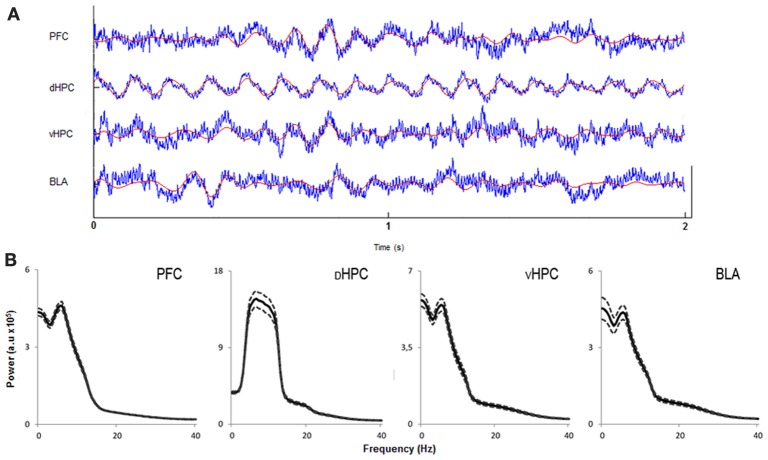
**Characterization of local field potentials recorded in the OF arena. (A)** Representative traces from local field potentials simultaneously recorded from the medial prefrontal cortex (PFC), dorsal hippocampus (dHPC), ventral hippocampus (vHPC), and basolateral amygdala (BLA) in one rat exploring the OF arena during the first exposure. Raw traces are plotted in blue and filtered theta traces (5–12 Hz) are overlayed in red. Presented segment duration is 2 s. Voltage scale (bottom right) is −0.2 to 0.2 mV for PFC, vHPC, and BLA; and −0.4 to 0.4 mV for dHPC. **(B)** Power spectra for PFC, dHPC, vHPC, and BLA. Spectra are average of multitaper spectrum estimates for all animals (*n* = 14) during the first exposure to the OF arena within the 10–25 cm/s speed range. Dotted lines are ± s.e.m. a.u, arbitrary units.

In order to assess how global theta activity of the HPC-BLA-mPFC circuit varied in response to habituation to a novel arena, theta power from LFPs recorded from electrodes in validated positions in the dHPC, vHPC, BLA, and mPFC (see Figure S1 for histological validation) in freely moving rats during exposures to the OF was analyzed. Robust theta oscillations during exploratory activity were observed in LFPs recorded from the dHPC in accordance with previous studies (McFarland et al., 1975; Hinman et al., 2011). Theta oscillations with equal robustness, but lower magnitude, were also observed in the vHPC, mPFC, and BLA during exploration. Although theta oscillations in the vHPC, mPFC, and AMY have been shown to occur less frequently than in the dHPC (Adhikari et al., 2010; Royer et al., 2010; Lesting et al., 2011) this may depend on behavioral task or status. Our observations fit with previous studies showing the emergence of robust theta activity in these areas during exploration (Royer et al., 2010; Patel et al., 2012), decision-making (Schmidt et al., 2013), anxiety (Adhikari et al., 2010), and fear (Lesting et al., 2011). Figure 3 shows representative traces of simultaneously recorded LFPs from the mPFC, dHPC, vHPC, and BLA and respective power spectra where theta activity is visible in all brain areas.

**Figure 4:**
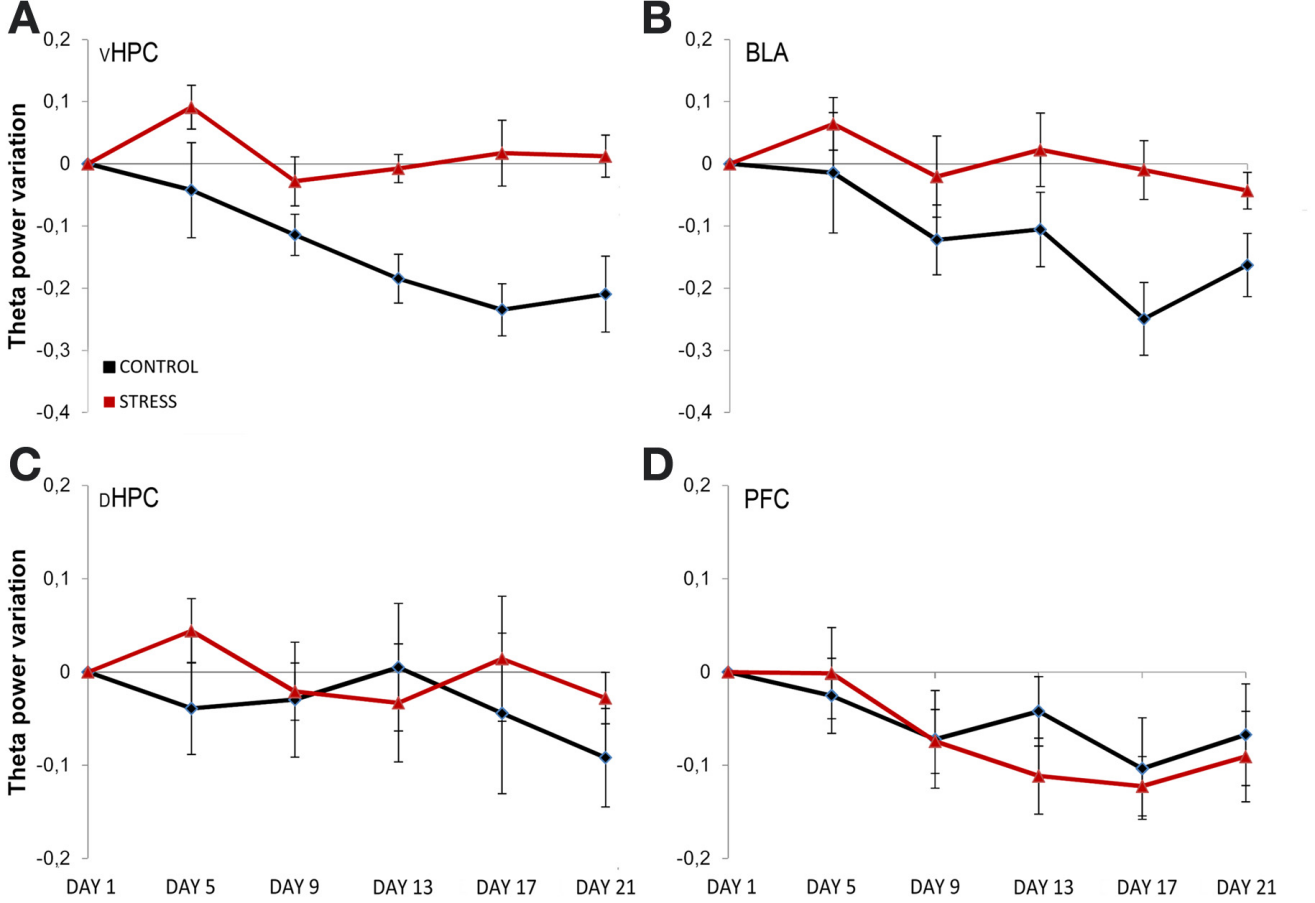
**Temporal evolution of theta power variation from initial exposure with familiarization.** Theta power variation during all exposures to the OF arena (day 5, day 9, day 13, day 17, and day 21) relative to theta power in the initial exposure (day 1, origin) from activity recorded from ventral hippocampus (vHPC) **(A)**, basolateral amygdala (BLA) **(B)**, dorsal hippocampus (dHPC) **(C)**, and medial prefrontal cortex (PFC) **(D)**. Theta power decreased in the vHPC and BLA in control rats with familiarization; the same decrease was not observed in stressed rats. No differences were observed between the groups for dHPC and PFC theta power variation with familiarization despite a trend for PFC theta power decrease on both groups. Also note that the second exposure to the OF arena (which corresponded to 4 days of exposure to stressors on stress group rats) showed an incremental effect of stress on theta power as vHPC activity increased from the first exposure on stressed rats only. Error bars: ± s.e.m.

To assess the temporal dynamics in theta power during familiarization with the arena, theta power variation from the first exposure (day 1, origin) to the following exposures (day 5, 9, 13, 17, and 21, respectively) were analyzed (Figure 4). Theta power variation between the first day of exposure and the following exposures was calculated as described in the methods section. Controls and stressed rats presented two opposing temporal patterns in vHPC and BLA theta power: while there was a continuous decrease of vHPC and BLA theta power with familiarization in controls, stressed rats showed no variation of the same measure in respect to the initial exposure during the same time period (Figures 4A,B). mPFC theta power also had the tendency to decrease with familiarization but followed a similar pattern in both groups. The second exposure to the OF arena (day 5) also appeared to reveal an incremental effect of stress on theta activity: stressed rats showed increased theta power in the dHPC, BLA and especially vHPC.

**Figure 5:**
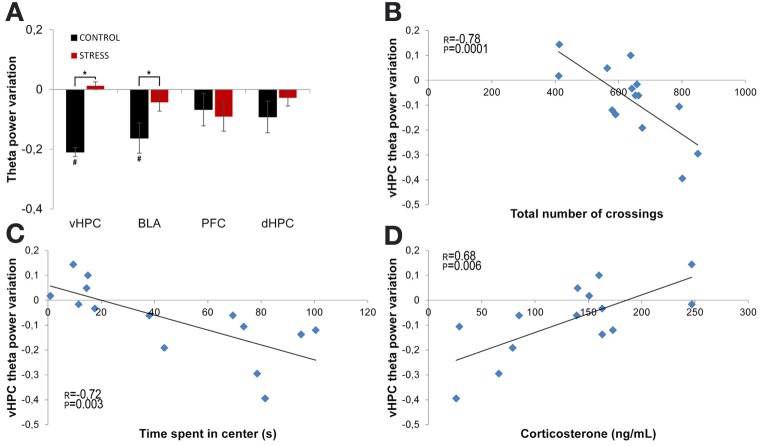
**Theta power variation between first and last exposures to the OF arena. (A)** Theta power variation between the initial exposure (unfamiliar environment, day 1, origin) and the final exposure (familiar environment, day 21) to the OF arena from activity recorded from ventral hippocampus (vHPC), basolateral amygdala (BLA), dorsal hippocampus (dHPC), and medial prefrontal cortex (PFC). Control rats significantly reduced theta power in vHPC and BLA through familiarization between the first and last exposure to the OF arena while stressed rats failed to display a similar reduction. **(B)** Correlation of vHPC theta power variation between first and last exposures to the OF arena and exploratory activity, as measured by the total number of crossings between the squares on which the arena was divided for behavioral analysis, on the last exposure to the OF arena. **(C)** Correlation of vHPC theta power variation between first and last exposures to the OF arena and time spent in the center on the last exposure to the OF arena. Rats that presented the biggest reduction of vHPC theta power between the first and last exposure to the OF arena were the same that presented higher exploratory activity and spent more time in the center on the last exposure to the arena. **(D)** Correlation of vHPC theta power variation between first and last exposures to the OF arena and serum corticosterone measured on the day following the last exposure to the arena. Rats that presented the lowest levels of serum corticosterone on the day following the last exposure to the OF arena were also the same that presented the highest reduction of vHPC theta power between the first and last exposure to the OF arena. #*p* < 0.05 for paired Wilcoxon sign rank test comparison of within group theta power estimates between exposures; ^*^p < 0.05 for unpaired Wilcoxon rank sum test comparison of average theta power variation between groups on each exposure. Error bars: ± s.e.m. Plotted *r* and *p* values for correlations are for Pearson correlation.

Theta power variation between the first day of exposure to the OF (novel arena) and the last day of exposure (familiar arena) was then compared (Figure 5). The comparison between day 1 (first exposure, origin) and day 21 (sixth and final exposure) revealed that control animals presented a significant decrease of vHPC and BLA theta power and no global variation of dHPC and mPFC theta power (vHPC *p* = 0.03; BLA *p* = 0.007; Figure 5A). In contrast, stressed animals presented an absence of significant global theta power variation in all brain areas during the same time period (Figure 5A). In other words, stressed animals failed to display the decrease in theta power in the vHPC and BLA observed in controls upon familiarization with the OF. As a result, when theta power variations were compared between controls and stressed animals there were significant differences in the vHPC and BLA, but not in the dHPC or mPFC (vHPC: *p* = 0.04; BLA: *p* = 0.03).

Interestingly, vHPC theta power variation between the first and last day of exposure to the OF arena was significantly, but negatively, correlated with behavioral measures of exploratory activity and anxiety-like behavior in the OF measured on the last day of exposure (correlation of vHPC theta power variation with total distance travelled: *r* = −0.78, *p* = 0.0001, Figure 5B; correlation of vHPC theta power variation with time spent in center: *r* = −0.72, *p* = 0.003; Figure 5C). Animals which had a higher reduction of vHPC theta power between the first and last day of exposure to the OF also travelled the most distance and spent the most time in the center in the final exposure day. Noticeably, theta power variation in the vHPC between the first and last day of exposure to the OF arena was also significantly correlated (*r* = 0.68, *p* = 0.006) with serum corticosterone levels at the end of the stress protocol (Figure 5D). Rats exhibiting lower corticosterone levels presented a higher decrease in vHPC theta power and rats with higher corticosterone levels followed the opposite trend in vHPC theta power. dHPC, BLA, and mPFC theta power variation between the first and last exposure to the OF arena presented, for some of the behavioral parameters measured on the last day of exposure to the OF, some degree of correlation although much lower than the correlations observed for vHPC theta power and not reaching significance.

### Theta coherence variations in the limbic network during familiarization

**Figure 6:**
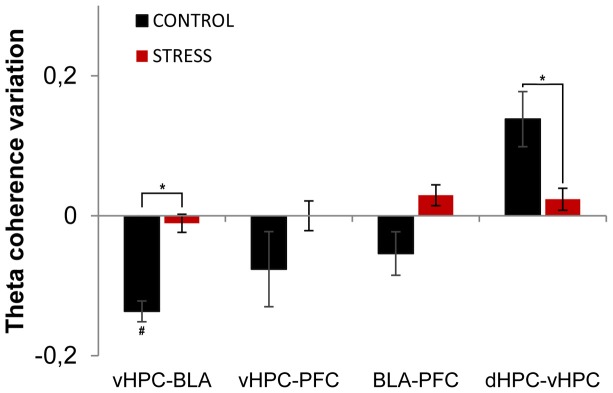
**Theta coherence variation between first and last exposures to the OF arena.** Theta coherence variation between the between the first exposure to the OF arena (unfamiliar environment, origin) and the final exposure to the same environment (familiar environment, day 21) for the following brain area pairs: vHPC-BLA, vHPC-PFC, dHPC-vHPC and BLA-PFC. Note that theta coherence variation presented for the novel environment is relative to theta coherence on the first exposure (day 1) to the OF arena. Controls presented a significant decrease of vHPC-BLA theta coherence and a tendency for decrease of vHPC-PFC and BLA-PFC theta coherence between first and last exposures to the OF arena; while stressed rats presented an absence of variation of theta coherence between the same brain area pairs. Control rats also presented increased dHPC-vHPC theta coherence through familiarization between first and last exposures to the OF arena while stress rats failed to display a similar variation. ^#^*p* < 0.05 for paired Wilcoxon sign rank test comparison of within group theta coherence estimates between exposures; ^*^p < 0.05 for unpaired Wilcoxon rank sum test comparison of average theta coherence variation between groups on each exposure. Error bars: ± sem.

Subsequently, we analyzed theta coherence variations between the first day and last day of exposure to the arena for pairs of recorded brain areas (Figure 6). Variation of theta coherence between the first and last day of exposure was calculated in a similar way to theta power (see Methods section). Longitudinal data analysis showed a significant decrease in vHPC-BLA theta coherence and a global trend for decrease in vHPC-PFC and BLA-PFC theta coherence with familiarization in controls (vHPC-BLA *p* = 0.04; vHPC-PFC *p* = 0.14; BLA-PFC *p* = 0.07, Figure 6), whereas in stressed animals no global variations were observed in theta coherence (Figure 6). As a result, when theta coherence variation was compared between control and stressed animals it differed significantly for vHPC-BLA and BLA-PFC theta coherence (vHPC-BLA *p* = 0.02; BLA-PFC *p* = 0.04).

Interestingly, dHPC-vHPC theta coherence had a tendency to increase between the first and last day of exposure to the arena on both groups, even though the increase was three times higher in controls (Figure 6). When comparing dHPC-vHPC theta coherence variation between control and stressed animals, a significant difference was found (*p* = 0.03).

In contrast, theta coherence variation between dHPC-PFC and dHPC-BLA presented no significant variation between exposures or between groups (dHPC-PFC: control 0.01 ± 0.02, stress 0.00 ± 0.02, control vs. stress *p* = 0.99; dHPC-BLA: control 0.00 ± 0.02, stress 0.00 ± 0.02, control vs. stress *p* = 0.73; values are mean ± sem).

### Re-exposure to a novel environment is correlated with changes in the vHPC-BLA network

We then explored how re-exposure to a novel unfamiliar arena would impact on theta activity within the vHPC-BLA circuit. For that purpose, all animals were exposed to a second novel environment which was an arena consisting of an enclosed space and an open space; the time animals spent in the closed space was taken as a measure of anxiety. Stressed animals spent 40% more time in the closed space than controls (*p* = 0.04).

**Figure 7:**
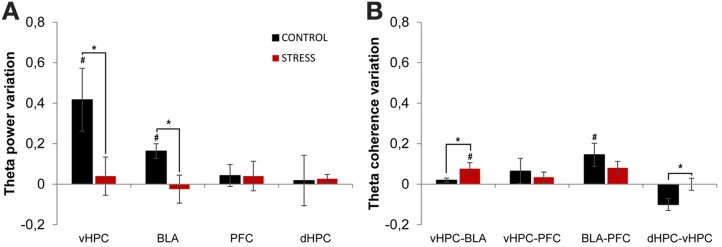
**Theta power and coherence variation between final exposure to the OF and novel unfamiliar environment. (A)** Theta power variation between last exposure to the OF arena (familiar environment) and the second novel unfamiliar arena from activity recorded from ventral hippocampus (vHPC), basolateral amygdala (BLA), dorsal hippocampus (dHPC), and medial prefrontal cortex (PFC). Note that theta power variation presented for the novel environment is relative to theta power in the last exposure (day 21) to the OF arena. When rats from both groups were exposed to the new unfamiliar arena a significant theta power increase in vHPC and BLA was observed in controls but not on stressed rats. No significant variations were observed in dHPC and PFC theta power between plotted exposures or between groups on each exposure. **(B)** Theta coherence variation between the last exposure to the OF arena (familiar environment, day 21) and the second novel unfamiliar arena (day 24) for the following brain area pairs vHPC-BLA, vHPC-PFC, dHPC-vHPC, and BLA-PFC. When all rats were moved to the new unfamiliar environment control rats presented a significant increase of BLA-PFC theta coherence only; while stressed rats presented a significant increase of vHPC-BLA theta coherence. On dHPC-vHPC theta coherence, control rats presented a decrease whereas stressed rats showed no variation. #*p* < 0.05 for paired Wilcoxon sign rank test comparison of within group theta power and theta coherence estimates between exposures; ^*^p < 0.05 for unpaired Wilcoxon rank sum test comparison of average theta power and theta coherence variation between groups on each exposure. Error bars: ± s.e.m.

Theta power activity in the dHPC, vHPC, mPFC, and BLA and theta coherence between brain areas was compared between the last day of exposure to the OF (familiar environment, day 21) and the second novel arena (novel unfamiliar environment, day 24) (Figure 7). Control animals presented a significant theta power increase in vHPC and BLA, but no significant variation of dHPC and mPFC theta power (vHPC *p* = 0.04; BLA: *p* = 0.04; Figure 7A). In contrast, stressed animals presented an absence of significant global theta power variation in all brain areas under analysis (Figure 7A). As a result, when mean theta power variation was compared between experimental groups, it differed significantly in vHPC and BLA, but not in dHPC or mPFC (vHPC *p* = 0.03; BLA *p* = 0.03).

We then analyzed theta coherence variation between brain areas under study (Figure 7). Noticeably, controls presented a significant increase in BLA-PFC theta coherence when moved to the novel arena, but not on any other inter-regional combinations, despite a trend for reduction of dHPC-vHPC theta coherence (BLA-PFC *p* = 0.03; dHPC-vHPC *p* = 0.13, Figure 7B); on the contrary, stressed animals presented only a significant increase in vHPC-BLA theta coherence (vHPC-BLA *p* = 0.01; Figure 7B). When variation in theta coherence was compared between control and stressed animals it differed significantly for vHPC-BLA and dHPC-vHPC pairs only (vHPC-BLA *p* = 0.03; dHPC-vHPC *p* = 0.01; Figure 7B), being lower for controls in both cases.

### Speed cannot account for observed variations in theta power

Theta power has been described to increase with running speed (McFarland et al., 1975), most prominently in the septal pole of the dHPC (Hinman et al., 2011). To test if the variation in theta power observed herein could be accounted for by speed modulation the correlation between mean theta power and mean speed of all segments within the 10–25 cm/s speed range for each brain area and each animal was calculated. Pearson correlation coefficients were averaged across animals for the same brain regions. Both on the first and last day of exposure to the OF arena a small correlation of dHPC theta power with speed was observed (dHPC day 1: *r* = 0.13 ± 0.01; dHPC day 21: *r* = 0.10 ± 0.01). This correlation was significant for more than half of the animals. However, vHPC, mPFC, and BLA theta power showed no significant correlation with speed (vHPC day 1: *r* = 0.02 ± 0.02; mPFC day 1: *r* = 0.00 ± 0.02; BLA day 1: *r* = 0.02 ± 0.02; vHPC day 21: *r* = −0.02 ± 0.01; mPFC day 21: *r* = 0.02 ± 0.02; BLA day 21: *r* = 0.02 ± 0.02). Similar correlation values were found in the re-exposure to the novel unfamiliar environment.

## Discussion

### Theta activity modulation in the vHPC and BLA is relevant for environmental risk assessment behavior

An environment which has no salient threats but nevertheless increases the possibility of threat occurrence, like the OF arena and especially its central region, triggers in rats a state of generalized anxiety that is characterized by defensive quiescence and exploratory risk assessment (Grey and McNaughton, 2003). This risk assessment prevails as long as safety-directed active avoidance behavior is relatively inhibited, which is likely to occur due to the absence of imminent threats, and allows the gathering of more information from the environment or possible threat while the simultaneous preparation of the animal for a potential flight-or-fight response occurs (Steimer, 2002; Behrendt, 2011). Establishing familiarity with such an environment contributes to the reduction of the anxiety triggered by it (Blanchard et al., 2011). Thus, when a rat is first exposed to a novel environment it is expected that the circuits regulating anxiety-related defensive behaviors including risk assessment and passive avoidance increase their activity. Likewise, when the environment becomes familiar it is expected that the activity of these circuits diminishes, especially the ones regulating avoidance and the inhibition of exploration, and that a return to normal behavior occurs (Blanchard et al., 2011). One of such circuits involves the ventral hippocampus (vHPC) and the amygdala (AMY, in particular its basolateral component—BLA), with outputs to the mPFC. The hippocampus has been suggested to integrate and compare information from the neocortical sensory areas with internal states and take over control of behavior when a possible threat (or a mismatch between input from the environment and internal expectation) is detected (Grey and McNaughton, 2003), thus playing a crucial role in novelty-induced exploratory behavior and familiarization (Manahan-Vaughan and Braunewell, 1999; Eichenbaum et al., 2007). The amygdala, although historically related to fear, also plays a role in novelty-induced exploration and anxiety-like behavior, most likely through its connection with the vHPC, as it conveys important processed threat stimulus to the hippocampus heightening arousal (McGaugh, 2005) and modulating affective memory (Roozendaal et al., 2009). Indeed, lesions of the vHPC and BLA reduce anxiety-like behavior across various behavioral tests (Deacon et al., 2002; Bueno et al., 2005).

Spectral power from LFPs has been used as an indicator of regional activity and theta power in particular has been a measure consistently used to infer on theta oscillations specific activity from the HPC and other areas to which the hippocampus is connected (Buzsáki, 2002). Theta oscillations are believed to be involved in the temporal synchronization of hippocampal neurons, altering for example plasticity, and between the hippocampus and downstream areas such as the AMY or PFC (Buzsáki, 2002; Seidenbecher et al., 2003; Jones and Wilson, 2005). Consistent with the roles of the HPC and AMY in anxiety and with the role of theta oscillations as a synchronization process, we found herein that familiarization with the environment over the course of 3 weeks was correlated with a significant reduction of theta power in the vHPC and BLA, but not in the dHPC and mPFC, in control rats. That this reduction occurred in the vHPC and not in the dHPC is not surprising as there is a growing body of evidence confirming the existence of functional dissociation along the septotemporal axis of the hippocampus (Fanselow and Dong, 2010). Functionally, the dHPC has been more closely related with learning, spatial memory and navigation tasks, while the vHPC has a preferential role in emotional and affective behaviors, namely in anxiety-like behavior including risk-assessment. This dissociation arises from the fact that the vHPC is more closely connected with the mPFC, AMY, and bed nucleus of stria terminalis (BNST) (Behrendt, 2011), all key structures in anxiety, fear and defensive behaviors and stress response. The similar reduction of theta power observed in the BLA is also not surprising as the amygdala is implicated in the processing of threat stimulus and defensive behaviors (Phelps and LeDoux, 2005). Besides the co-activation of the vHPC and BLA, a general trend for decrease of theta coherence was observed within the vHPC-BLA-mPFC circuit, suggesting that a decrease of synchronized activity within this network was correlated with the process of familiarization. Importantly, and consistent with the above mentioned hypothesis, is the fact that controls significantly increased the time spent in the center of the OF between the first and last exposure to the arena, thus displaying a phenotype related with a reduction of generalized anxiety.

The decrease of vHPC theta power in the final exposure to the OF when compared with the initial exposure 3 weeks before, was highly correlated with the time rats spent in the center of the arena. Rats that presented the highest decrease of theta power in the vHPC after 3 weeks of familiarization with the OF were the ones that spent the most time in the center in the final exposure day. Moreover, and confirming that the exposure to a novel environment is associated with hyperactivation of vHPC and BLA, when the same rats that were allowed to familiarize with the OF arena were placed in a novel environment a significant increase in theta power was observed again in vHPC and BLA, but not in dHPC and mPFC. Whether the variations in activity described are only neuronal hallmarks of the anxiety-driven behavior (and its extinction) in the brain areas that modulate anxiety behavior or are themselves regulating the said behavior, is still an open question and theta disruption studies would be needed to clarify this issue. Nevertheless, previous studies (Grey and McNaughton, 2003; Gordon et al., 2005; Adhikari et al., 2010), as well as our data, show that theta frequency oscillations in the hippocampus are tied with and play a role in the expression of anxiety.

### Chronic stress impairs theta activity modulation of the vHPC and BLA

In contrast to controls, in stressed animals no reduction of theta power in the vHPC and BLA during familiarization to the OF arena was observed. This is indicative that the hyperactivation of vHPC and BLA was maintained over the course of the 3 weeks in stressed animals. Overactivation of vHPC and BLA by stress has been previously described in works on brain slices (Rainnie et al., 2004; Maggio and Segal, 2009) and anaesthetized rats (Kavushansky and Richter-Levin, 2006) including in the theta range (Oliveira et al., 2013) and appears to be in accordance with the dendritic hypertrophy observed in neurons of these brain regions after chronic stress exposure (Mitra et al., 2005), as a result of persistent hypercortisolism. Persistent hyperactivation of the amygdala with concurrent loss of inhibitory drive of the dHPC and the mPFC is also tied with the maladaptation of chronic stress through the contribution to the persistent overactivation of the HPA axis (Flandreau et al., 2012). Obviously, the relevance of increased theta activity observed in the vHPC into the BNST-paraventricular nucleus of the hypothalamus (PVN) function is more complex to integrate.

The longitudinal analysis of the variations in theta activity during the initial period of familiarization revealed that repeated exposures to stressful events have an incremental effect in theta power in the vHPC and BLA during the first days of stress exposure. In fact, while on the second exposure to the OF (which corresponded to 4 days of stress exposure) controls did not display any variation in theta power from the first exposure, stressed rats had further increases in theta power in the vHPC and BLA. Interestingly, the analysis of the subsequent exposure to the arena (third exposure) showed that controls already presented a decrease of theta power in both vHPC and BLA when compared with the first exposure, although of lower magnitude when compared with the final exposure. This suggests that the decrease of activation of those regions through familiarization in controls occurred around the third exposure; contrarily, a similar adaptation was not found in stressed rats.

In terms of behavior, persistent hyperactivation of vHPC and BLA in stressed rats was correlated with increased defensive immobility and reduced approach to the most aversive region of the OF. This chronic stress-induced phenotype has been previously described (Katz et al., 1981). The data from the present study suggests that repeated exposures to an initially novel environment without proper modulation of vHPC-BLA activity correlates with increased averseness of that environment tipping the scale to the avoidance side of the exploratory conflict and impairing a return to normal non-defensive behavior. Unlike controls, stressed rats did not increase the time spent in the center of the OF arena and greatly decreased the overall exploratory activity over the course of 3 weeks, thus suggesting the presence of some inhibitory defensive drive directing them toward the safer region of the arena and inhibiting overall exploration.

Theta power variation in the vHPC between the first and last exposures to the OF arena was strongly and negatively correlated with the levels of serum corticosterone of each rat at the end of the stress protocol. Rats that presented the lowest decrease of theta power between exposures (stressed rats) were also the ones that had the highest levels of corticosterone at the end of the experimental protocol (this also correlated with the time animals spent in the center of the OF as previously described). It is unclear at this stage if it were corticosterone levels that modulated theta activity or if they are just a hallmark of stress where anxious behavior and anxiety-driven neural activity are expected. However, it is well known that hypercortisolism generated by prolonged stress exposure affects the hippocampus both morphologically and functionally. Morphologically, prolonged exposure to corticosteroids triggers atrophy of neurons in the hippocampus (Sousa and Almeida, 2012). In terms of synaptic plasticity, high doses of corticosterone can impair hippocampal CA1 long-term potentiation (LTP) (Diamond et al., 1992). However, it is important to note that the majority of studies on the effects of corticosteroids on hippocampal activity were done in the dHPC and their effects along the hippocampal axis, including the vHPC, are not entirely known. Previous reports suggest that dHPC and vHPC synpaptic plasticity reacts differently to stress (Hawley et al., 2012; Maggio and Segal, 2012) Curiously, this differential response led to the recent proposal that corticosterone may act as a switch, regulating the routes by which the HPC is preferentially linked to the rest of the brain (Maggio and Segal, 2012). According to this view, under stress, the vHPC route to the AMY and other limbic/subcortical structures traditionally involved in emotional responses is enhanced, while the dHPC route to the neocortex, involved in higher-order cognitive functions, is reduced. In line with this perspective, a significant increase of vHPC-BLA theta coherence during the re-exposure to a novel environment was observed only in stressed animals. It is also well known that the amygdala plays a role in hippocampal plasticity both under normal and stressful conditions (Roozendaal et al., 2009), suggesting that inputs from the amygdala to the hippocampus are an important signal to the comparator function of the hippocampus (Grey and McNaughton, 2003) and to the switching to the limbic route under stress that underlies behavioral disruption.

An additional interesting finding is that the possible switching of hippocampal function to the limbic/subcortical route appears to also affect intra-hippocampal theta synchronization. In fact, controls presented an increase in dHPC-vHPC theta coherence with familiarization three times higher than the one observed for stressed rats; curiously, when control animals were re-exposed to a novel environment there was a trend for a decrease in dHPC-vHPC theta coherence that rendered the difference between groups significant. Noticeable are the facts that amygdala-mediated hippocampal LTP decreases with chronic stress (McEwen, 2007) and that dHPC-vHPC theta coherence was described to be inversely correlated with vHPC-PFC theta coherence, a measure pertaining to anxiety in the EPM (Adhikari et al., 2010).

Stress has been extensively shown to reduce brain plasticity and behavioral flexibility. The observation that stressed animals cannot modulate theta activity of the vHPC and BLA in response to familiarization or when re-exposed to a novel environment reinforces the view that these transitory modulations are critically implicated in the appropriate adjustments of risk assessment and arousal behaviors in different contexts.

## Methods

### Animals

A total of 14 Male Wistar-Han rats (Charles River laboratories, Barcelona, Spain), weighing 300–350 g and aged 12 weeks at the time of surgery were used in this study. Animals were single-housed under the following laboratory conditions: room temperature 22°C, relative humidity of 55%, 12 h light cycle beginning at 8 am, food and water *ad libitum*. Experiments were conducted in accordance with European Union Directive 86/609/EEC and the Portuguese regulations and laws on the protection of animals used for scientific purposes of the Ministry for Agriculture, Rural Development and Fishing. This study was approved by Veterinary General Direction (DGV).

### Surgery

Following a period of two weeks of handling for at least once a day for 5 min, animals were subjected to a surgery for implantation of chronic single-wire electrodes. Electrodes were assembled in-house from formvar insulated nichrome single wires (Science Products GmbH, Hofheim, Germany), 50 μm inner diameter (66 μm outer diameter), tightly wrapped around golden Mill-Max receptacles (Mill-Max Mfg. Corp., Oyster Bay, NY, USA). Animals were kept under anesthesia during the whole procedure with a gaseous mixture of 2–4% sevoflurane in 100% oxygen. Electrodes were implanted, through burr-holes, and targeted the mid-ventral portion of the pre-limbic area of the prefrontal cortex (3.3 anterior, 0.8 lateral, and 4.0 depth), the dorsal portion of the hippocampus (3.9 posterior, 2.2 lateral, and 2.4 depth), the ventral portion of the hippocampus (4.8 posterior, 4.8 lateral, and 8.4 depth), and the BLA (2.4 posterior, 4.9 lateral, and 8.6 depth). A stainless-steel screw electrode over the cerebellum (10.5 posterior, 0.0 lateral) served as ground. All electrodes were cemented directly to the skull and connected to a Mill-Max connector. The final assembly was cemented with dental acrylic resin (GC America Inc., Alsip, IL, USA), with four additional skull screws serving as anchors. Animals were allowed to recover for 15 days.

### Stress protocol

After recovery from surgery animals were familiarized with the recording room and tethering procedures. Briefly, the animals were transported in their home cage to the recording room where they were left alone for 5 min. They were then tethered to the recording system via a flexible cable headstage and allowed to freely behave in their home cage during another 15 min. This procedure was repeated for 4 days. The animals were then divided in two groups: a control group (*n* = 7) and a stress group (*n* = 7). The stress protocol started after the habituation period ended and was carried for 20 days. The CUS protocol has been described elsewhere (Cerqueira et al., 2007) and is known to induce a sustainable stress response throughout the entire period of exposure to the protocol (Pêgo et al., 2008). Briefly, stress group animals were exposed to a daily stressor (up to 1 h a day). In order to avoid adaptation, the same stressor was never applied in consecutive days and the hour of the day at which the stressor was applied was chosen randomly. Four different stressors were used: restraint, noise, shaking and cold air stream. All stressors were applied in a separate experimental room from where the animals of both groups were housed. The control group was handled for the same time during the same period.

### Open field/familiar arena

On the day the stress protocol started, animals from both groups were introduced to a novel OF arena in the recording room. The OF is widely used to assess explorative behavior, reaction to novelty and anxiety among other parameters (Ennaceur et al., 2006). The OF used in this experiment consisted of a medium-density fiberboard (MDF), gray-colored, square arena with each side measuring 60 cm and with walls 50 cm high.

After being tethered to the recording system, animals were placed in the center of the arena and allowed to freely behave for 15 min. To study the effects of chronic stress on the familiarization to the novel arena, this procedure was repeated two times a week for the entire duration of the stress protocol in a total of six exposures for both experimental groups. There were always 3 days of interval between recording sessions. All recordings took place in the morning (9:00–12:00 am). In recording days, including on the first day of exposure to the OF arena, stress group animals were only exposed to the daily stressor after the recording session, in order to minimize any acute stress response. The sixth and final exposure to the arena took place the day after the stress protocol ended (day 21).

### Efficacy of the stress protocol

On the day following the last exposure to the OF arena blood samples were drawn from all animals. Blood samples were collected by tail venipuncture in the morning (9:00–12:00 am). The samples were centrifuged at 13000 rpm for 10 min. Serum was extracted and stored at −80°C for posterior analysis. Serum corticosterone levels were measured using 125I radioimmunoassay (RIA) kits (MP Biomedicals, Inc., Orangeburg, NY, USA). Reduced or slow body weight gain has also been associated with the efficacy of stress protocols (Pêgo et al., 2008) therefore, body weights were recorded on a weekly basis and body weight gain between the start and end of the stress protocol was calculated for each animal.

### Exposure to novel unfamiliar environment

Following one day of rest after the blood samples were collected, the rats were introduced to a novel unfamiliar arena in which they were allowed to freely behave for 15 min.

### Behavioral analysis

Behavioral analysis was done automatically in a matlab-based platform developed in our laboratory for behavioral and LFPs integrated analysis in freely moving rodent's experiments. Briefly, the OF floor was divided in 16 equal squares. The four central squares were considered as the center and the remaining squares as periphery. Animal's position was given by the video-tracking system. Total time in each region of the arena was equal to the total time each animal spent on squares corresponding to the center or the periphery. Total distance travelled was calculated as the total number of crossings between squares.

### Data acquisition

Signals were acquired during behavior in the arenas in single-ended non-referenced mode using the dacqUSB system (Axona Ltd., London, UK) at 24 kHz. Field potential signals were amplified and low-pass filtered with a 600 Hz cut-off frequency. A 50 Hz notch filter was applied in all recordings. Position coordinates were also acquired (at 20 Hz) with an integrated video-tracking system from an infra-red LED on the headstage connected to the animal's headmount.

### Data analysis

Data were imported into Matlab (Mathworks, Natick, MA, USA) and analyzed with custom-written code that uses Chronux toolbox (http://www.chronux.org) (Mitra and Bokil, 2008). Data was first downsampled to 1.2 kHz and detrended using the function *locdetrend* from the Chronux toolbox.

Theta power estimates were calculated with a multitaper method using Chronux. The window size was 0.6 s with an overlap of 50%. The time-bandwidth product (TW) was chosen as 3 and the number of tapers (K) was 5. Frequency resolution was chosen to be 0.6 Hz. Total theta power on each window was obtained by averaging the spectral power estimates of all frequencies in the 5–12 Hz band. Theta spectral coherence between all brain regions was calculated for the same windows using similar multitaper parameters.

Windows with movement artifacts or signal saturation were removed from posterior analysis. Mean speed was also calculated for each window by averaging all speed values within the window. Speed was calculated as the distance between two consecutive tracking positions obtained by the video-tracking system. In order to avoid variations due to possible modulation of theta power by speed and to discard periods where the animal was immobile or in rearing activity, only windows that had a mean speed that fell inside the 10–25 cm/s range were used in the final analysis. In order to assess any possible modulation of theta power by speed in the windows used for analysis, Pearson correlation coefficients (matlab function *corr*) were calculated between speed and theta power estimates for each brain area.

Theta power variation between the first exposure (day 1, origin) and the following exposures (days 5, 9, 13, 17, and 21, respectively) to the OF and between the final exposure to the OF (day 21) and the second novel unfamiliar arena (day 24) were given by the ratio of theta power estimates of the later recording minus the earlier recording divided by the earlier recording. This was calculated for each animal and then averaged across animals within each group. A similar procedure was used for theta coherence variation. This normalization measure, for both theta power and theta coherence, takes the first exposure to the OF (day 1) as origin and is positive if theta power or coherence increases with familiarization, negative if it decreases and takes a value of zero if unaffected by familiarization (Figures 4A, 6). Since the same procedure is also applied to the comparison between the final exposure to the OF (day 21) and the second novel arena (day 24) (Figure 7), it is important to note that, in this case, the origin will be the final exposure to the OF (day 21) and the measure is positive if theta power or coherence increases in the novel arena in relation to the final exposure to the OF, negative if it decreases and zero if no variation between environments is present.

### Histology

To confirm the position of the electrodes, at the end of the experimental period, all animals were deeply anesthetized with pentobarbital (100 mg/Kg). An electrolytic lesion was done by passing current through all the electrodes. The animals were then perfused transcardially with fixative (4% paraformaldehyde). The brains were removed and placed in fixative solution. After further fixation the brains were coronally sectioned in 45 μm slices, collected on non-coated glass slides, stained with Giemsa and mounted with Entellan-New (Merck, Darmstad, Germany). Electrode tip position was confirmed by microscopic observation of the slides (Figure S1).

### Statistics

All datasets were tested for normality using the Shapiro-Wilk test. Since the majority of datasets did not exhibit normal distributions or the significance of the normality assumption was low, Wilcoxon sign rank test (*signrank* function from matlab) was used to compare power and coherence estimates from the same group at different time points. Wilcoxon rank sum test (*ranksum* function from matlab) was used to compare power and coherence estimates between stress and control groups. Results are expressed as mean ± standard error of the mean (s.e.m.).

Linear correlations between theta power variation and corticosterone and behavioral parameters were measured by Pearson linear correlation coefficient.

### Conflict of interest statement

The authors declare that the research was conducted in the absence of any commercial or financial relationships that could be construed as a potential conflict of interest.

## Supplementary material

Figure S1 **Histological confirmation of electrodes' positions. (A)** Diagrammatic coronal sections of medial prefrontal cortex (mPFC) (left), basolateral amygdala (BLA) (middle-left), dorsal hippocampus (dHPC) (middle-right), ventral hippocampus (vHPC) (right). Sections are arranged from most anterior (top) to most posterior (bottom). Electrolytic lesion sites are marked as green crosses. PL, prelimbic area of PFC; IL, infralimbic area of the mPFC; BLA, basolateral amygdala. Diagrammatic coronal sections are adapted from Paxinos and Watson (2006). **(B)** Representative histological sections showing electrolytic lesions in mPFC (left), dHPC (middle-left), vHPC (middle-right), and BLA (right). Black arrows mark electrode position.
