# Supplementary figures and images for: Stress affects theta activity in limbic networks and impairs novelty-induced exploration and familiarization
Source: Front Behav Neurosci. 2013 Oct 14;7:127. doi: 10.3389/fnbeh.2013.00127 (PMC3797543; doi:10.3389/fnbeh.2013.00127)

A

PFC

BLA

dHPC

vHPC

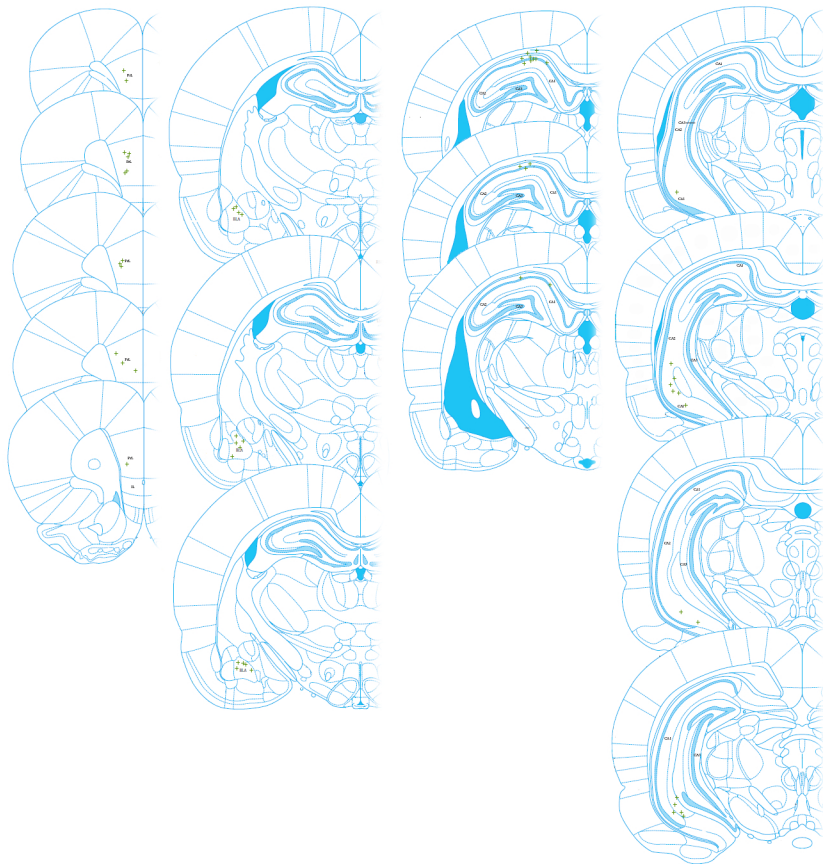

B

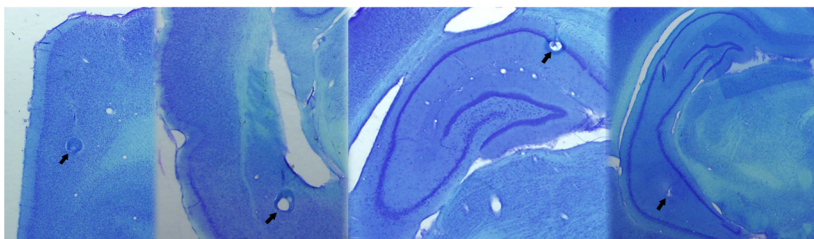

Supplement: Figure S1 — Histological confirmation of electrodes' positions. (A) Diagrammatic coronal sections of medial prefrontal cortex (mPFC) (left), basolateral amygdala (BLA) (middle-left), dorsal hippocampus (dHPC) (middle-right), ventral hippocampus (vHPC) (right). Sections are arranged from most anterior (top) to most posterior (bottom). Electrolytic lesion sites are marked as green crosses. PL, prelimbic area of PFC; IL, infralimbic area of the mPFC; BLA, basolateral amygdala. Diagrammatic coronal sections are adapted from Paxinos and Watson (2006). (B) Representative histological sections showing electrolytic lesions in mPFC (left), dHPC (middle-left), vHPC (middle-right), and BLA (right). Black arrows mark electrode position. [file DataSheet1.pdf]
